# Cardiovascular Disease in Anti-neutrophil Cytoplasm Antibody-Associated Vasculitis

**DOI:** 10.1007/s11926-023-01123-8

**Published:** 2023-11-28

**Authors:** Matthew Sayer, Gavin B. Chapman, Matthew Thomas, Neeraj Dhaun

**Affiliations:** 1 Edinburgh Kidney, University/BHF Centre for Cardiovascular Science, The Queen’s Medical Research Institute, University of Edinburgh, Edinburgh, UK; 2 Department of Renal Medicine, Royal Infirmary of Edinburgh, Edinburgh, UK

**Keywords:** ANCA vasculitis, Cardiovascular disease

## Abstract

**Purpose of Review:**

Anti-neutrophil cytoplasm antibody (ANCA)-associated vasculitis (AAV) is a rare, multisystem, autoimmune disease characterised by microvascular inflammation. Over the past 20 years, advances in immunological management have improved short-term patient outcomes. Longer-term patient outcomes remain poor with cardiovascular disease now the leading cause of death in AAV. Here, we examine the potential pathways that contribute to the increased risk of cardiovascular disease in AAV and the current evidence to manage this risk.

**Recent Findings:**

The incidence of cardiovascular disease in AAV exceeds that expected by traditional risk factors alone, suggesting a contribution from disease-specific factors. Similarly, it is unclear how different immunosuppressive therapies contribute to and modify cardiovascular risk, and there is a paucity of data examining the efficacy of traditional cardioprotective medications in AAV.

**Summary:**

There is a lack of evidence-based cardiovascular risk assessment tools and cardioprotective therapies in patients with AAV which should be addressed to improve long-term outcomes.

## Introduction

Cardiovascular disease is defined by the World Health Organisation and European Heart Network as an umbrella term encompassing all disorders that affect the heart muscle and circulatory system [1, 2]. These include, but are not limited to, coronary heart disease, cerebrovascular disease, and rheumatic heart disease. Cardiovascular disease is the commonest non-infectious cause of death globally. Between 1990 and 2019, the number of people dying annually of cardiovascular disease rose from 12.1 to 18.6 million worldwide [3]. Cardiovascular disease imposes a huge economic burden; it is reported to cost the European Union economy €210 billion annually, equating to 8% of total healthcare expenditure [4]. Consequently, there is an increasingly urgent need to address the rising incidence of cardiovascular disease and to reduce its impact on both patients and healthcare services globally.

Since its inception in 1948, the Framingham Heart Study has identified several cardiovascular risk factors including increasing age, smoking, hypertension, and diabetes mellitus [5]. More recent is the recognition that autoimmunity is also associated with elevated cardiovascular risk [6]. Anti-neutrophil cytoplasm antibody (ANCA)-associated vasculitis (AAV) is a prototypic autoimmune disease. The impact of cardiovascular disease in AAV is significant. Indeed, it is now the major cause of death for patients with AAV over the longer term. [7]. In this article, we review the factors contributing to the increased rates of cardiovascular disease in AAV and the evidence base for modifying cardiovascular risk in this patient group.

## Anti-neutrophil Cytoplasm Antibody-Associated Vasculitis

AAV is a rare autoimmune disease characterised by inflammation of the small and medium blood vessels which leads to endothelial cell injury and end-organ damage [8]. It has an overall prevalence of approximately 400 per million people globally [8] and comprises a group of three separate, but overlapping, conditions: granulomatosis with polyangiitis (GPA), microscopic polyangiitis (MPA), and eosinophilic granulomatosis with polyangiitis (EGPA). All three are defined by a loss of tolerance to neutrophil granule proteins, typically either proteinase-3 (PR3) or myeloperoxidase (MPO) [9]. Whilst most patients have circulating autoantibodies to these leucocyte antigens, 5–10% of patients are ANCA-negative. The average age of disease onset is 50–70 years old with men and women affected equally. There is significant geographical variation in the prevalence of GPA which is typically more common with increasing latitude. Additionally, the incidence of GPA is higher in populations of primarily European ancestry, whereas MPA is more common in East Asian populations [8].

**Fig. 1:**
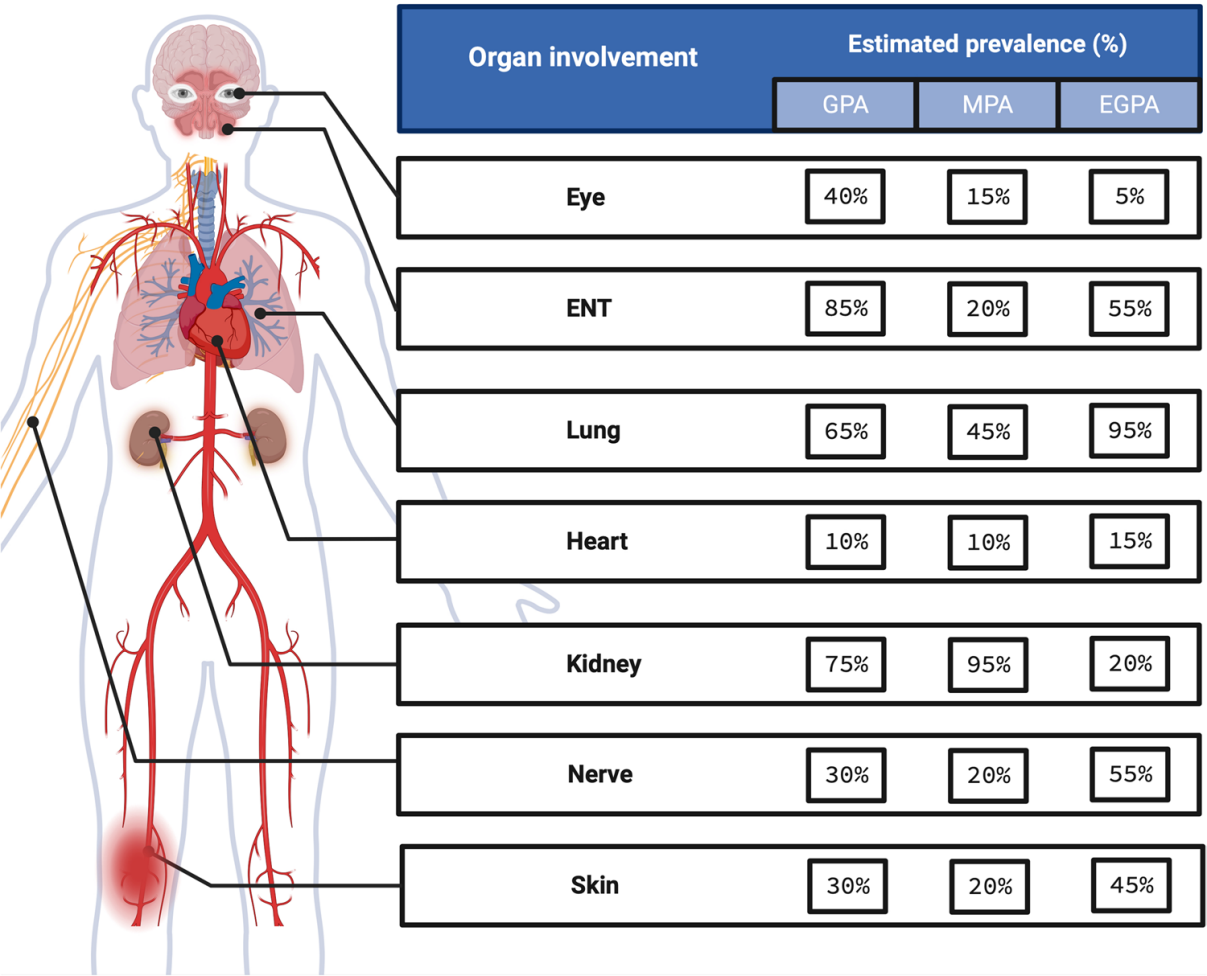
Published frequencies of specific organ system involvement in GPA, MPA, and EGPA. These data are taken from an analysis of 673 subjects with newly diagnosed AAV enrolled into one of five different prospective, randomised clinical trials conducted by the European Vasculitis Study Group [10]. Percentages for EGPA are based on 511 patients from three studies analysed in an evidence-based guideline of the diagnosis and management of EGPA [11]. GPA, granulomatosis with polyangiitis; MPA, microscopic polyangiitis; EGPA, eosinophilic granulomatosis with polyangiitis; AAV, anti-neutrophil cytoplasm antibody-associated vasculitis

AAV can be difficult to diagnose as the condition can affect any organ system and presenting symptoms can vary between patients. All three conditions share non-specific symptoms of chronic inflammation such as arthralgia, fatigue, myalgia, and weight loss. Organs that are commonly involved in AAV include the upper and lower respiratory tract, eyes, lungs, kidneys, and skin. GPA, MPA, and EGPA each have typical patterns of organ involvement although there is significant phenotypic heterogeneity and overlap such that diagnostic classification can be challenging (Fig. 1) [8]. In addition, these diseases can present with variable tempo including indolent presentations that are often associated with significant diagnostic delay through to rapidly progressive and fulminant presentations that may be life-threatening (e.g., pulmonary haemorrhage or rapidly progressive glomerulonephritis). Once a diagnosis of AAV is confirmed, the immediate goal of treatment is to rapidly suppress inflammation and limit organ damage with immunosuppressive therapies (‘remission induction’) [12]. Once remission is achieved, ongoing immunosuppressive treatment (‘remission maintenance’) is usually required to prevent recrudescence of disease activity (‘flare’ or ‘relapse’).

Without treatment, mortality from AAV is ~80% within 12 months [13]. Since the introduction of immunosuppressive therapies in the 1960s, the short-term prognosis has improved dramatically and 5-year survival is now 70–80% [14]. Unfortunately, long-term prognosis has not improved to the same extent. Cardiovascular disease is now the major cause of death in patients with AAV in the longer term [7]. MPO-positive AAV is associated with a higher risk of cardiovascular disease than PR3-positive AAV, which may reflect different patterns and severity of organ involvement at diagnosis [15].

## Pathogenesis of AAV and Cardiovascular Disease

**Fig. 2:**
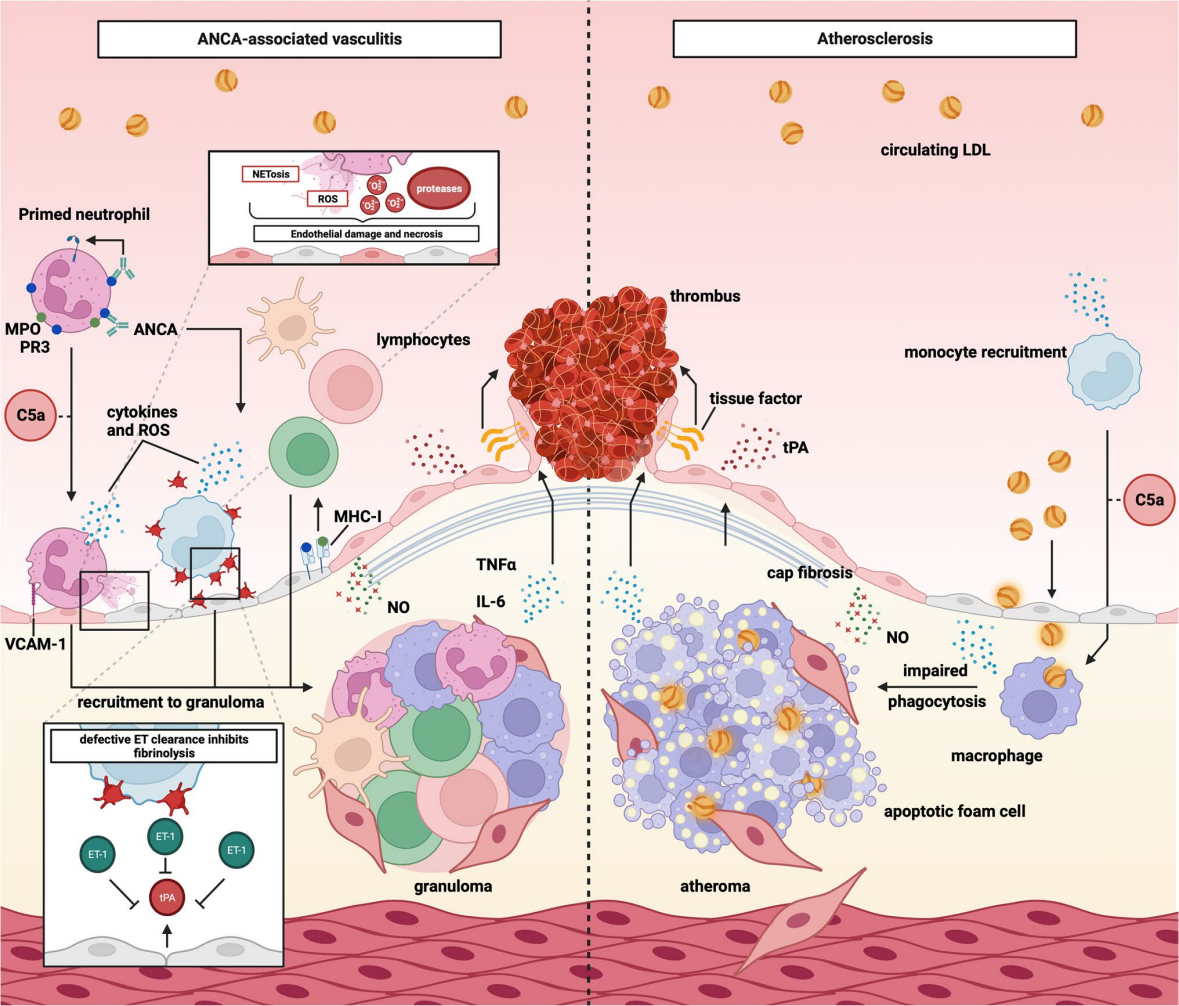
Anti-neutrophil cytoplasm antibody-associated vasculitis and atherosclerosis involve shared cellular pathways. Both conditions are characterised by activation of monocytes, macrophages, and the complement system as well as production of inflammatory cytokines and reactive oxygen species. The net result is an increase in circulating LDL-cholesterol and endothelial dysfunction. The latter is characterised by a reduction in nitric oxide production and an increase in endothelin-1 production by endothelial cells. This results in arterial stiffening and a reduction in endothelium-dependent vasodilatation. The inflammatory cytokines and endothelin-1 are prothrombotic and result in reduced production of tissue plasminogen activator which reduces clot breakdown. MPO, myeloperoxidase; PR3, proteinase 3; C5a, complement component 5a; ROS, reactive oxygen species; VCAM-1, vascular cell adhesion molecule; MHC-1, major histocompatibility complex class 1; NO, nitric oxide; TNF-α, tumour necrosis factor-alpha; IL-6, interleukin-6; tPA, tissue plasminogen activator; ET-1, endothelin-1; LDL, low-density lipoprotein

Central to AAV pathogenesis is loss of B- and T-cell tolerance to the neutrophil proteins PR3 or MPO displayed on the cell surface of primed neutrophils. Priming occurs in the presence of pro-inflammatory mediators (e.g., interleukin-1β, interleukin-6, and tumour necrosis factor-α) or due to complement activation via the alternative pathway [8]. B-cells produce autoantibodies that bind to MPO/PR3 on the primed neutrophil cell surface leading to neutrophil activation [8]. Activated neutrophils perform many crucial steps in the pathogenesis of AAV. They *1)* adhere to the vascular endothelium; *2)* degranulate and release pro-inflammatory cytokines and proteases; *3)* generate reactive oxygen species; and *4)* expel neutrophil extracellular traps (NETs) and microparticles [8]. Alongside neutrophils, ANCA activates monocytes and macrophages [16], further contributing to microvascular inflammation and promoting thrombosis through interactions with platelets [17]. The endpoint of these processes is endothelial cell injury and necrosis. Progressive inflammation and cell death lead to fibrosis with a resultant loss of organ function (Fig. 2).

Endothelial injury is also central to the development of atherosclerosis [18]. A critical early step in atherogenesis is deposition of circulating low-density lipoprotein cholesterol (LDL-C) within the vascular intima that occurs due to impaired endothelial barrier function [18]. This triggers monocyte recruitment and activation, with subsequent differentiation into macrophages which take up LDL-C. These ‘foam macrophages’ accumulate within the intima leading to atheroma progression and macrophage apoptosis. Ordinarily, apoptotic cells are cleared by phagocytosis or efferocytosis but the sheer number of foam cells, coupled with impaired macrophage function, leads to the development of a necrotic core at the centre of this atherosclerotic plaque (Fig. 2) [18]. Ongoing inflammation leads to plaque instability and eventual rupture. The exposed contents of the necrotic core interact with circulating blood cells to promote thrombus formation. Fragmentation of the thrombus can lead to a distal infarct.

There are several shared mechanisms in the pathogenesis of AAV and atherosclerosis that may, in part, explain the elevated cardiovascular risk in patients with AAV (Fig. 2). First, endothelial dysfunction is critical to both diseases. In health, the endothelium plays a key role in vascular tone and haemostasis through the production of nitric oxide and tissue plasminogen activator (tPA) which promote vasodilatation and fibrinolysis, respectively [19]. These effects are countered by the production of endothelin-1 (ET-1), the most potent endogenous vasoconstrictor [20]. Endothelial dysfunction is associated with an imbalance in favour of ET-1 and against nitric oxide and tPA. Indeed, a recent study demonstrated that patients with AAV in long-term clinical remission (median 2.4 years) have a two-fold higher plasma ET-1 concentration and impaired endothelial tPA release compared to age- and sex-matched healthy subjects [21]. Similarly, impaired fibrinolysis is a hallmark feature of atherosclerotic cardiovascular disease [22].

Second, an increase in circulating LDL-C concentration positively associates with the development of atherosclerotic cardiovascular disease in the general population [23]. Patients with AAV have been shown to develop elevated LDL-C following diagnosis [24], possibly due to a combination of the treatments they are given and endothelial dysfunction, which has itself been associated with increases in proprotein convertase subtilisin/kexin type 9 (PCSK9) concentrations in those with kidney impairment [25]. PCSK9 is an important regulator of tissue LDL-receptor expression. Circulating PCSK9 binds to cell surface LDL-receptors promoting lysosomal degradation leading to a rise in circulating LDL-C. PCSK9 expression increases during systemic inflammation [26].

A third shared mechanism involves monocyte activation and recruitment. In AAV, monocyte activation persists even once disease remission has been achieved and this may contribute to subclinical chronic inflammation [16] which is a recognised contributor to the development and progression of atherosclerosis [27]. Monocyte recruitment to the site of endothelial injury in AAV or into the intima in atherosclerosis leads to impaired efferocytosis of apoptotic macrophage cells. Dysfunctional cell clearance is possibly driven by release of proteases from neutrophils and contributes to release of pro-inflammatory cytokines which are known to cause further endothelial cell damage in AAV or promote LDL-C migration in atherosclerosis [16, 18].

A final common mechanism is activation of the alternative complement pathway. This process is particularly topical due to the recent licencing of the C5a inhibitor, avacopan, for the treatment of AAV [28]. The complement pathway is active in many autoimmune diseases [29], and there is evidence from other autoimmune conditions linking C5a activity to future cardiovascular risk [30, 31]. C5a also plays a role in cell adhesion and monocyte migration to the intima in atherosclerosis [32]. Thus, one might anticipate that avacopan use in AAV improves long-term cardiovascular outcomes in these patients, and we look forward to the real-world data that might support or refute this hypothesis.

## Cardiovascular Risk in Patients with AAV at Diagnosis

There are limited data on the prevalence of traditional cardiovascular risk factors in patients with AAV at diagnosis. Although several randomised controlled trials have focused on the early immunological management of AAV over the last 20 years [28, 33–38], their reporting of cardiovascular risk factors has been limited to age and sex (Table 1). Thus, these data are largely derived from observational studies. A recent retrospective cohort study compared 1,520 patients with a new diagnosis of AAV with 5,834 controls matched for age, sex, kidney function, income, rurality, and number of hospitalisations in the preceding three years [39]. The authors found no difference in rates of hypertension or diabetes mellitus between the groups; unfortunately, they did not report on smoking status, lipids, or body mass index. In another study, Berti et al. retrospectively identified 58 patients with AAV and found that, at diagnosis, patients had lower total cholesterol concentrations compared to 174 age- and sex-matched controls [40]. However, these data are limited by the fact that lipid values could have been taken up to 3 years before or after the diagnosis of AAV was made. A sub-analysis of serum samples from 142 patients in the Rituximab for ANCA-associated Vasculitis (RAVE) trial showed total cholesterol and LDL-C increased in the 6 months following diagnosis, particularly for patients with a new diagnosis or those with PR3-positive AAV [24]. This change was seen for both rituximab and cyclophosphamide treated patients and occurred independent of glucocorticoid exposure or change in BMI. Based on these findings, it is recommended that repeat measures of lipids are performed with lipid-lowering treatment initiated thereafter as necessary.

**Table 1:** Reporting of cardiovascular risk factors in randomised controlled trials in AAV

Trial	Study population	Intervention	Primary outcome	CV risk factors reported in patients at baseline	CV events reported	Follow up
Non-renal GPA Treated Alternatively with MTX (NORAM, 2005)	New diagnosis of AAV (*n* = 100)	CYC + GC versus MTX + GC	Remission at 6 months	Age Sex	1 presumed cardiac death. No adverse CV events reported	18 months
PEX for renal vasculitis (MEPEX, 2007)	New diagnosis of AAV with sCr >512 μmol/L (*n* = 137)	IV MP versus PEX	Renal recovery at 3 months	Age Sex	9 CV adverse events (not further defined)	12 months
Pulsed versus daily oral CYC in AAV (CYCLOPS, 2009)	New diagnosis of AAV with renal involvement (*n* = 149)	Pulsed CYC versus daily CYC	Time to disease remission	Age Sex	None reported	18 months
Rituximab versus CYC in AAV with renal involvement (RITUXVAS, 2010)	New diagnosis of AAV with renal involvement (*n* = 44)	Rituximab + GC versus CYC + GC	Sustained remission and severe adverse events at 12 months	Age Sex	2 CV deaths. No other adverse CV events reported.	12 months
Rituximab *versus* CYC for AAV (RAVE, 2010)	New diagnosis or relapse of AAV (*n* = 197)	Rituximab + GC versus Cyc + GC	Remission at 6 months	Age Sex	None reported	6 months
PEX and glucocorticoids in severe AAV (PEXIVAS, 2020)	New diagnosis or relapse of AAV with kidney or lung involvement (*n* = 704)	PEX or no PEX with standard GC regimen or reduced GC	Composite of death from any cause or ESKD	Age Sex	124 CV events (not further defined) reported in appendix	12 months
Avacopan for the treatment of AAV (ADVOCATE, 2020)	New diagnosis or relapse of AAV (*n* = 331)	Avacopan vs. GC	Disease remission at 6 months and sustained remission at 12 months	Age Sex BMI	1 death from myocardial infarction. 157 CV events.	12 months

*AAV*, ANCA-associated vasculitis; *BMI*, body mass index; *CV*, cardiovascular; *CYC*, cyclophosphamide; *ESKD*, end-stage kidney disease; *GC*, glucocorticoid; *IV*, intravenous; *MP*, methylprednisolone; *MTX*, methotrexate; *PEX*, plasma exchange; *sCr*, serum creatinine

A larger analysis was recently reported which examined the association between autoimmunity more broadly and cardiovascular risk [6]. Here, the authors compared 446,449 patients diagnosed with an autoimmune disease (e.g., 66,796 patients diagnosed with rheumatoid arthritis and 37,940 patients diagnosed with vasculitis (including temporal arteritis, giant cell arteritis, polyarteritis nodosa, and AAV)) with 2,102,830 controls, matched for age, sex, socioeconomic status, and region. The study had several important findings. At the point of diagnosis, there was no difference between patients diagnosed with an autoimmune condition and the control group with regards to blood pressure, body mass index, or lipid profile (assessed by total cholesterol to high-density lipoprotein ratio). Over a median follow-up of 6.2 years, the risk of cardiovascular disease was increased in all 19 autoimmune diseases studied to a similar extent to that seen in patients with an existing diagnosis of type 2 diabetes mellitus. This increased risk was greatest in conditions associated with inflammation, autoantibody-mediated pathology, and endothelial dysfunction, all features of AAV. Finally, the authors concluded this excess risk could not be explained by traditional cardiovascular risk factors alone but was due to autoimmunity *per se*.

Previously, cardiovascular risk was thought to be highest in the year following a diagnosis of AAV, a time when disease is most active and the focus of treatment is achieving disease remission [39]. However, a recent nested case-control study of patients in Denmark analysed cardiovascular outcomes in the year preceding the diagnosis of AAV for 2,371 patients diagnosed between 1996 and 2021. Each patient was matched to three age- and sex-matched control subjects. The study found that cardiovascular disease rates were elevated throughout the 12 months preceding diagnosis, with risk peaking in the month immediately prior to diagnosis [41]. It is well recognised that the diagnosis of AAV is often delayed [8], and the unopposed inflammation and endothelial injury prior to diagnosis are probably important contributors to this increased cardiovascular disease risk.

## Effects of AAV Treatment on Cardiovascular Risk

There are several treatment options available for AAV depending on disease presentation. However, our understanding of their differential effects on cardiovascular risk is limited as clinical trials have only recently begun to report cardiovascular events during follow-up (Table 1).

### Glucocorticoids

Glucocorticoids remain the mainstay of treatment for AAV [42]. Their use across a range of diseases has been associated with increased cardiovascular morbidity and mortality [43, 44]. Exogenous glucocorticoid use is associated with the development of diabetes mellitus, dyslipidaemia, hypertension, and weight gain, side effects mediated through the development of insulin resistance, salt and water retention, and direct effects on endothelial function and vascular smooth muscle tone [45].

A recent UK-based study in 87,794 patients diagnosed with at least one immune-mediated inflammatory disease demonstrated a dose-dependent increase in incident cardiovascular disease; even patients taking <5 mg of prednisolone per day experienced a 1.7-fold increased risk of all-cause cardiovascular disease compared to those who were not taking regular glucocorticoids [46]. Another study analysed long-term data from four European Vasculitis Study Group trials of patients with GPA and MPA [47]. Of the 296 patients for whom medication data were available, 147 patients received glucocorticoids for at least three years following a diagnosis of AAV. Duration of glucocorticoid use associated with the development of hypertension and irreversible organ damage, both of which link to increased mortality [48]. However, given that patients with more active disease at presentation may be treated with higher- and longer doses of glucocorticoids, the results from this study may be confounded.

Recent trials in AAV have aimed to reduce glucocorticoid burden [38, 49]. The Low-Dose Glucocorticoid Vasculitis Induction Study (LoVAS) randomised 140 patients with AAV to receive rituximab in combination with either standard (initially 1 mg/kg/day; total dose ~4.2 g) or reduced-dose (0.5 mg/kg/day; total dose ~1.3 g) prednisolone [49]. There were no differences between groups with respect to disease remission at six months and, encouragingly, the reduced-dose group had lower rates of diabetes mellitus and infection with a trend towards less dyslipidaemia (12% versus 17%). Disappointingly, the findings of LoVAS with respect to cardiovascular outcomes were not replicated in the PEXIVAS (Plasma Exchange and Glucocorticoid Dosing in Severe AAV) trial which similarly compared a standard glucocorticoid taper (~3.2 g oral glucocorticoid therapy in the first 3 months) versus reduced-dose (~1.8 g) with and without plasma exchange in patients with AAV [38]. Encouragingly, PEXIVAS did show that reduced-dose glucocorticoid was non-inferior to standard-dose glucocorticoid for remission-induction when given alongside cyclophosphamide and/or rituximab. It is also possible that the higher rates of cardiovascular disease seen in PEXIVAS were due to the fact that the trial only studied patients with severe AAV, defined as an estimated glomerular filtration rate <50 ml/min/1.73 m^2^ or diffuse pulmonary haemorrhage. Nonetheless, these trials have paved the way towards reduced dose glucocorticoid regimens in AAV becoming standard of care [42].

### Rituximab

Rituximab, a monoclonal antibody initially developed as a treatment for B-cell non-Hodgkin lymphoma, selectively depletes CD20 expressing B-cells [50]. Rituximab is now standard-of-care in many parts of the world, both for inducing disease remission in AAV and as part of maintenance immunosuppression to prevent disease relapse. Experimental studies in mice have demonstrated a protective effect of anti-CD20 therapy with regard to atherosclerosis development and cardiac remodelling following myocardial infarction [51, 52].

There are few clinical data relating rituximab use to cardiovascular disease risk. A large retrospective cohort study of 1,602 patients with pemphigus, an autoimmune disease affecting the skin and mucosal surfaces, compared safety outcomes in patients treated with rituximab (*n* = 801) versus mycophenolate mofetil or azathioprine (*n* = 801) [53]. Rituximab appeared cardioprotective, with these patients having lower rates of myocardial infarction, stroke, and peripheral vascular disease, and less likely to develop hypertension, hyperlipidaemia, and type 2 diabetes mellitus. However, this study did not provide data on concomitant medications and so the difference in cardiovascular outcomes may relate to other factors (in particular, between-group differences in glucocorticoid therapy).

In kidney transplantation, rituximab use has been associated with lower rates of atherosclerotic cardiovascular disease at eight years follow-up [54]. Again, this study did not account for glucocorticoid therapy, a mainstay of kidney transplantation immunosuppression. Finally, there is evidence in patients with rheumatoid arthritis suggesting rituximab has beneficial effects on endothelial function [55]. However, this study is limited by its small size, lack of comparator group, and enrolment of almost exclusively female patients. Overall, high-quality evidence from clinical trials regarding rituximab therapy and cardiovascular outcomes is lacking.

### Cyclophosphamide

Cyclophosphamide, an alkylating agent that induces changes in DNA structure and cell death *via* apoptosis, was initially developed in the 1950s as an anti-cancer drug but is now used in a range of autoimmune diseases, including AAV. In patients with cancer, cyclophosphamide therapy has been associated with heart failure, myocarditis, and pericarditis [56], and this risk appears dose-dependent [57]. The CYCAZAREM (Cyclophosphamide versus Azathioprine for Early Remission Phase of Vasculitis) trial compared oral cyclophosphamide with azathioprine as maintenance therapy in AAV [58]. Long-term follow-up (median 8.5 years) data from this randomised controlled trial of 144 patients found six patients in the cyclophosphamide group suffered a cardiovascular death versus one patient in the azathioprine group (*p* = 0.11) [59]. There are no other data linking cyclophosphamide use in AAV with incident cardiovascular disease. Given it has been the mainstay of AAV induction treatment for many years and is often the active comparator (either with or without rituximab) in trials of emerging therapies, it would be of value to determine the impact of cyclophosphamide use in AAV on long-term cardiovascular outcomes through collaborative acquisition of real-world data or those from pharma-sponsored studies.

### Plasma Exchange

Plasma exchange (PEX) removes plasma and the immunoglobulins circulating within it and is recommended as a treatment option for AAV in patients with severe kidney involvement (serum creatinine >300 μmol/L) [42]. PEXIVAS, the largest randomised controlled trial in patients with AAV to date, enrolled 704 patients with new or relapsing AAV with kidney involvement or diffuse alveolar haemorrhage and randomised them to PEX or no PEX, alongside glucocorticoids and rituximab and/or cyclophosphamide [38]. Over a median follow-up of 2.9 years, rates of serious cardiovascular adverse events were no different between the two groups. Similarly, the MEPEX (Plasma Exchange for Renal Vasculitis) trial which randomised patients with AAV to PEX or intravenous methylprednisolone showed no difference in rates of cardiovascular disease between groups at 12 months follow-up, although reported event rates were low [35]. Longer-term follow-up from MEPEX (~4 years) also showed no difference in cardiovascular mortality between groups [60].

Intriguingly, the American Society for Apheresis recommends PEX as a treatment option for familial hypercholesterolaemia as it can lower LDL-C concentrations by ~50% [61]. However, this recommendation is based on low-quality evidence and, as PEX also removes high-density lipoprotein cholesterol and fibrinolytic factors, it is unclear whether the LDL-C-lowering effect translates to improved outcomes. There are also several risks with PEX including infection, bleeding, and line-related complications [62].

### Azathioprine and Mycophenolate Mofetil

Azathioprine inhibits purine synthesis and is used as part of maintenance immunosuppression in AAV. The RITAZAREM (Rituximab as Therapy to Induce Remission After Relapse in ANCA-Associated Vasculitis) trial was designed to assess whether fixed-interval rituximab (1 g every four months for five doses) was superior to azathioprine (2 mg/kg/day for 24 months) as maintenance immunosuppression in patients with relapsing AAV [63]. One hundred and seventy patients were randomised and 141 completed 48 months follow-up. Rather surprisingly, the trial reported no cardiovascular safety events.

Data from the MAINTAIN Nephritis (Azathioprine *Versus* Mycophenolate Mofetil for Long-Term Immunosuppression in Lupus Nephritis) trial, a randomised study in 105 patients with lupus nephritis (median follow-up 48 months), reported one episode of angina and one episode of cerebrovascular disease in the group treated with azathioprine compared to no events in the mycophenolate mofetil (MMF) group [64]. Given both AAV and SLE are both associated with elevated cardiovascular disease risk, the event rates reported in these studies seem unreasonably low.

MMF also acts by inhibiting purine synthesis and is used in the immunological management of patients with AAV. The MYCYC (Mycophenolate Mofetil versus Cyclophosphamide for the Induction of Remission in Nonlife-Threatening Relapses of ANCA-Vasculitis) trial compared MMF to pulsed cyclophosphamide in 140 patients with newly diagnosed AAV [65]. There were no differences in rates of cardiovascular disease between the two groups over 18 months follow-up. In an animal model of SLE, MMF lowers blood pressure [66]; supportive clinical data are limited to a small observational study of eight patients with autoimmune disease and hypertension (five patients with rheumatoid arthritis; three patients with psoriasis) which showed that MMF reduced systolic, diastolic, and mean arterial blood pressure, effects that were abolished on discontinuation of the drug [67]. Interestingly, there is evidence from *in vitro* and animal studies that MMF may have anti-atherogenic properties through effects on T-lymphocytes, monocytes, and vascular smooth muscle cells [68, 69], but clinical studies are lacking.

## Effects of AAV Disease Remission on Cardiovascular Risk

A study of 24 patients with small and medium vessel vasculitis (14 with GPA, seven with polyarteritis nodosa, and three with EGPA) demonstrated that treatment-induced disease remission is associated with a reduction in microvascular inflammation and improved endothelial function [70]. Similarly, in a study of 31 patients with AAV (15 patients with active disease), those in clinical remission had reduced arterial stiffness compared to patients with active disease [71]. Both endothelial dysfunction and increased arterial stiffness are independently associated with future cardiovascular disease risk [72, 73].

Although cardiovascular risk may fall once patients have achieved disease remission, it remains elevated. Farrah et al. recruited 32 patients with AAV in long-term disease remission (median 2.4 years) and 32 age- and sex-matched healthy subjects [21]. Despite most of the patients receiving optimal cardioprotection (aspirin, blockers of the renin-angiotensin system, and statins), they demonstrated impaired endothelial function and increased arterial stiffness similar in magnitude to patients with advanced kidney disease or those who have recently suffered a myocardial infarction. Importantly, in this study, AAV patients had no history of cardiovascular disease, and their kidney function was normal suggesting that the elevated cardiovascular risk was a disease legacy. These findings highlight the importance of screening and treating of modifiable cardiovascular risk factors in this patient group.

## Effect of AAV Disease Relapse on Cardiovascular Risk

Despite improvements in immunosuppressive regimens, AAV remains a relapsing and remitting disease with a relapse rate of ~50% by five years following diagnosis [8]. There are no studies that have specifically investigated the effect of relapse on cardiovascular risk. A disease relapse represents a recurrence of damaging inflammatory processes in patients with immunological memory. Disease relapse will lead to the accrual of organ damage with consequent patient frailty [15] and a likely increase in cardiovascular risk.

## Cardiovascular Risk Management in AAV

The European Alliance of Associations for Rheumatology (EULAR) recommends regular cardiovascular risk assessment in patients with AAV [42]. However, recommendations on optimal cardiovascular risk management are primarily based on expert opinion rather than randomised trials [74]. Also, there are currently no validated tools that accurately predict cardiovascular risk in AAV as they do in the general population (e.g., the Framingham Risk Score) [75].

There are few published data on how well cardiovascular risk is managed in AAV. Illustrating this point is an analysis of four EUVAS trials which included 270 patients with a new diagnosis of AAV and which reported no data on cardiovascular risk factor management. Some small-scale studies have reported on isolated risk factor management. For instance, a retrospective study from Germany assessed cardiovascular risk management in 53 patients with AAV [76]. The authors found 40 patients had at least a moderate risk of cardiovascular disease based on European Society of Cardiology guidelines, but only four had achieved recommended target LDL-C concentrations. Of the 43 patients with AAV and chronic kidney disease, only ~1 in 2 met guideline-based blood pressure targets. This may be due to inadequate recognition of the increased cardiovascular risk in these patients by clinicians, or because traditional evidence-based cardioprotective medications are less effective in AAV, or, more likely, due to a combination of these factors.

There are few data on prescriptions of cardioprotective medications in patients with AAV. A single-centre study of 32 patients with AAV in disease remission reported 69% of patients were receiving a blocker of the renin-angiotensin system, 60% of patients were on a statin, and 16% were prescribed aspirin [21]. However, this study excluded patients with known cardiovascular disease, chronic kidney disease, diabetes mellitus, hypertension, and/or dyslipidaemia prior to the diagnosis of AAV, therefore limiting the generalisability of these findings.

It may be that standard cardioprotective therapies are less effective in patients with AAV. Unfortunately, given the number of patients and duration of follow-up needed, alongside the lack of industry interest in off-patent medications, it is unlikely that these studies will ever be done. Data from small, retrospective studies suggest that aspirin may have a role in primary prevention of cardiovascular disease in autoimmune conditions [77, 78]. More recently, Conrad et al. showed that in a cohort of 446,449 patients with autoimmune disease in the UK, 8.5% of patients were prescribed aspirin [6]. It would be of interest to know whether in this large cohort aspirin use is associated with a lower risk of future cardiovascular events.

Whilst there are no data on the role of statin therapy in AAV, the JUPITER (Justification for the Use of Statins in Prevention: an Intervention Trial Evaluating Rosuvastatin) trial randomly assigned 17,802 patients with low serum LDL-C (<3.4 mmol/L) and elevated C-reactive protein concentrations (>2.0 mg/L) to rosuvastatin or placebo [79]. The trial was stopped early (after <2 years follow-up) due to the overwhelming benefits of rosuvastatin in reducing the rate of major cardiovascular events. Although this trial excluded patients on immunosuppressive therapy, the authors suggested statins may be beneficial in patients with low-grade inflammation. Encouragingly, in a recent meta-analysis of 12 studies (11 population-based cohort studies and one randomised controlled trial) including 148,722 patients diagnosed with an immune-mediated inflammatory disease and over 840,113 patient-years follow-up, statin therapy was associated with a reduction in all-cause mortality and major adverse cardiovascular events [80]. Despite this, only ~1 in 5 patients with autoimmune disease in the UK are on a statin [6].

## Looking to the Future

There is a need to develop and validate cardiovascular risk prediction tools not only for patients with AAV but for those with autoimmunity more broadly. Cardiac biomarker concentrations (e.g., high-sensitivity troponin) associate with future cardiovascular risk in the general population and chronic kidney disease [81, 82] and their utility in patients with AAV should be explored. Additionally, non-invasive cardiac imaging such as computed tomography coronary angiography (CTCA) is increasingly utilised to identify those patients at highest risk of future cardiovascular disease [83]. Specifically, coronary inflammation identified on CTCA strongly associates with future cardiovascular risk [84]. It would be interesting to see how this performs in AAV and how coronary inflammation changes, both in response to immunosuppressive treatment and over the disease course. Indeed, if fit-for-purpose, both cardiac biomarkers or CTCA metrics could be used as nested short- and longer-term cardiovascular endpoints in future clinical trials in AAV.

A research priority should be defining the effects of different immunosuppressive regimens on cardiovascular risk. Similarly, it is unclear how disease relapse modifies risk. It is encouraging that recent trials have started to report on cardiovascular events during follow-up (Table 1). If previous trials hold these data, alongside cardiovascular risk factor and outcome data, they should be released. These might help inform patient management where two different immunosuppressive regimens are considered equivalent from an immunological disease control perspective, but which may have varying cardiovascular benefits. Certainly, future clinical trials in this space should include cardiovascular events as a safety, and potentially efficacy, endpoint.

We need effective cardioprotective therapies, whether existing or new, for AAV. The STATVAS study [85] has completed recruitment; it is assessing whether rosuvastatin can reduce subclinical atherosclerosis in AAV. Its findings are eagerly awaited. Farrah et al. demonstrated short-term endothelin receptor antagonism improved endothelial function in patients with AAV in clinical remission [21]. The SPARVASC study [86] is currently assessing whether sparsentan (a dual endothelin and angiotensin receptor antagonist) can maintain this benefit longer-term.

Overall, to build upon the improvements in the immunological management of patients with AAV over the last 20 years, attention must now turn to the management and prevention of the cardiovascular complications of the disease to improve patient outcomes longer term.
